# Oxidative Stress and 4-hydroxy-2-nonenal (4-HNE): Implications in the Pathogenesis and Treatment of Aging-related Diseases

**DOI:** 10.1155/2022/2233906

**Published:** 2022-03-23

**Authors:** Yanling Li, Tingting Zhao, Jiaxin Li, Mengyao Xia, Yuling Li, Xiaoyu Wang, Chuanguo Liu, Tingting Zheng, Renjie Chen, Dongfang Kan, Yicheng Xie, Jingjie Song, Yu Feng, Tiangui Yu, Peng Sun

**Affiliations:** ^1^School of Pharmacy, Shandong University of Traditional Chinese Medicine, Ji'nan 250355, China; ^2^College of Foreign Languages, Shandong University of Traditional Chinese Medicine, Ji'nan 250355, China; ^3^Reproductive center, Affiliated Hospital of Shandong University of traditional Chinese Medicine, Ji'nan 250014, China; ^4^Innovation Research Institute of Chinese Medicine, Shandong University of Traditional Chinese Medicine, Ji'nan 250355, China; ^5^The Children's Hospital, Zhejiang University School of Medicine, National Clinical Research Center for Child Health, Hangzhou 310052, China; ^6^Shandong Mental Health Center, Ji'nan 250014, China

## Abstract

Oxidative stress plays an important role in the development of aging-related diseases by accelerating the lipid peroxidation of polyunsaturated fatty acids in the cell membrane, resulting in the production of aldehydes, such as malondialdehyde and 4-hydroxy-2-nonenal (4-HNE) and other toxic substances. The compound 4-HNE forms adducts with DNA or proteins, disrupting many cell signaling pathways including the regulation of apoptosis signal transduction pathways. The binding of proteins to 4-HNE (4-HNE-protein) acts as an important marker of lipid peroxidation, and its increasing concentration in brain tissues and fluids because of aging, ultimately gives rise to some hallmark disorders, such as neurodegenerative diseases (Alzheimer's and Parkinson's diseases), ophthalmic diseases (dry eye, macular degeneration), hearing loss, and cancer. This review aims to describe the physiological origin of 4-HNE, elucidate its toxicity in aging-related diseases, and discuss the detoxifying effect of aldehyde dehydrogenase and glutathione in 4-HNE-driven aging-related diseases.

## 1. Introduction

Aging is a natural phenomenon that all humans undergo, and delaying this process is a major challenge faced by the scientific community. Based on the research on aging mechanisms, mitochondria-derived free radicals are considered to play an important role in the process of cell senescence and the development of aging-related diseases. These mitochondria-derived free radicals include reactive oxygen species (ROS) [1], such as superoxide radicals (O_2_^•-^), H_2_O_2_, and OH^−^, as well as several reactive nitrogen species (RNS) [2]. An *in vivo* imbalanced ratio of antioxidant enzymes to ROS/RNS triggers an oxidative stress condition that consequently leads to oxidative protein modification [3], DNA damage due to altered methylation processes [4], and lipid peroxidation (LPO) of the cell membrane, thereby affecting cell proliferation, differentiation, apoptosis, and its related signal pathways. LPO of cellular membranes produces many secondary products, such as aldehydes and other toxic substances. Aldehydes, among which 4-HNE is the most toxic form, can accelerate the damage caused by oxidative stress. This mechanism of toxicity is primarily attributed to the binding of 4-HNE to protein, which inactivates some antioxidant enzymes and further aggravates oxidative stress, and ultimately apoptosis. Moreover, in a state of oxidative stress, cholesterol is another lipid compound that is susceptible to ROS attack which gives rise to an extensive series of molecules, termed oxysterols (7-ketocholesterol, 7*β*-hydroxy-cholesterol, 5*α*,6*α*- and 5*β*,6*β*-epoxycholesterol, and cholestan-3*β*,5*α*,6*β*-triol) [5, 6]. These damaging compounds can trigger the development of senile and neurodegenerative diseases [7] (such as Alzheimer's disease [AD], Parkinson's disease [PD], Huntington's disease [HD], and amyotrophic lateral sclerosis [ALS]), immune diseases, tumors [8], hearing loss [9], cardiovascular diseases (such as coronary heart disease [10]), and ophthalmic diseases (such as macular degeneration [11] and dry eye [12]).

With the recent technological advances such as the omics approach for redox proteins and detection of ROS-modified protein targets, clinical researchers can now better understand the mechanism of oxidative stress and aging-related diseases in the human body [13, 14]. Furthermore, improved 4-HNE-targeted detection technology and the application of multifunctional naturally occurring antioxidants have provided new research impetus for the treatment of 4-HNE-related oxidative stress diseases [15]. To date, antioxidants targeting the scavenging oxygen-free radicals have been identified to prevent the harmful effects due to oxidative stress. Aldehyde dehydrogenase (ALDH) and glutathione (GSH) have shown significant antioxidant effects on age-related diseases caused by ROS; hence, it is necessary to comprehensively explore the therapeutic potential of ALDH and GSH through in-depth experimental and clinical research [16–18]. The purpose of this review was to explain the physiological origin of 4-HNE, summarize its toxicity effects in aging-related diseases, and highlight the detoxification effects of GSH and ALDH.

## 2. Lipid peroxidation

In addition to ROS, lipids can be oxidized by enzymes like lipoxygenases, cyclooxygenases, and cytochrome P450. In particular, ROS are highly reactive and can rapidly react with lipids, proteins, and nucleotides. The accumulation of cellular ROS under oxidative stress conditions results in the induction of LPO and glycoxidation reactions, leading to the elevated endogenous production of reactive aldehydes and their derivatives such as glyoxal, methylglyoxal (MG), malondialdehyde (MDA), and 4-hydroxy-2-nonenal (4-HNE), which ultimately lead to advanced lipoxidation and glycation end products (ALEs and AGEs, respectively [19, 20]. MDA is the most mutagenic byproduct of LPO, whereas 4-HNE is the most toxic. Membrane phospholipids containing a large number of polyunsaturated fatty acids (PUFAs) are easily attacked by ROS and undergo LPO due to oxidative stress. Moreover, elevated intracellular iron concentration and a depletion of antioxidant GSH both increase ROS levels, consequently leading to ferroptosis, a form of cell death due to overwhelming membrane LPO [21–23]. LPO reactions in biofilms mainly occur through enzymatic and nonenzymatic pathways, whereby the nonenzymatic pathway is divided into two mechanisms: LPO mediated by either non-free radicals or free radicals [21]. In recent years, free radical-mediated nonenzymatic LPO has become the main focus of research on aging-related diseases because it leads to the formation of many secondary products, such as aldehydes, malondialdehyde, 4-HNE, and acrolein [24]. Because of their high reactivity and toxicity to the biological components of cells, these substances have attracted significant attention. Among them, 4-HNE is the most extensively researched, is frequently used as an oxidative stress marker [25, 26], can be used with oxysterols to assess oxidative stress [27, 28], and has been associated with the pathogenesis of cancer [29, 30], neurodegenerative diseases [31], diabetes [32], and other diseases.

## 3. 4-Hydroxy-2-nonenal

### 3.1. Sources of 4-HNE

The aldehyde compound 4-HNE is highly reactive and forms adducts with cellular proteins and DNA. It is also an effective signal molecule that regulates the mitogen-activated protein kinase (MAPK) pathway and the activity of redox-sensitive transcription factors (nuclear factor erythroid 2-related factor 2 [Nrf2], activating protein-1 [AP1], and NF*κ*B) [33], and influences several key signaling pathways, including the MAPK pathway, Jnk, and p38, PKC-*β* and *δ*, and Nrf2 [34].

**Figure 1:**
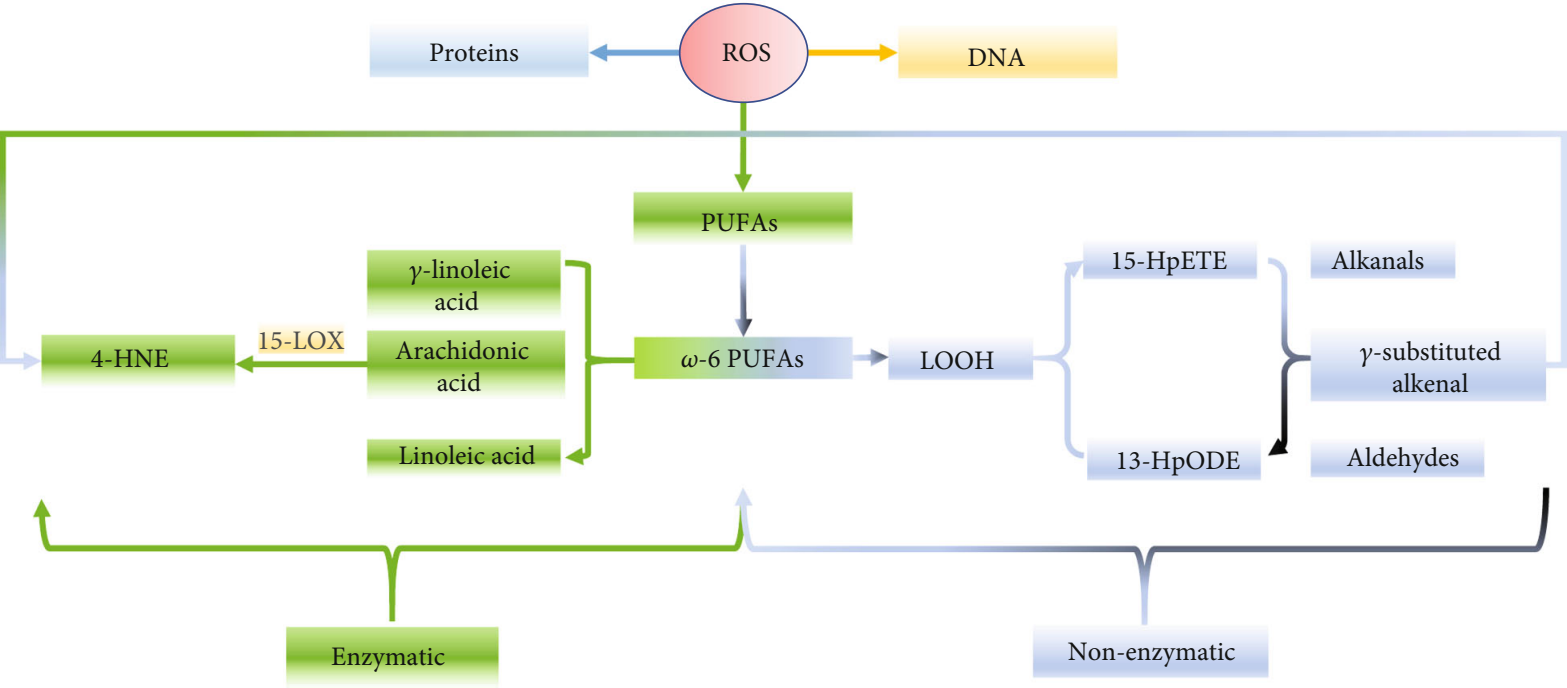
Production pathway of 4-hydroxy-2-nonenal (4-HNE). 4-HNE is produced by non-enzymatic and enzymatic pathways in *ω*-6 PUFAs induced by ROS.

A dyshomeostasis in ROS leads to the oxidative stress reaction of PUFAs, which can be divided into nonenzymatic and enzymatic pathways, as shown in Figure 1. In the nonenzymatic pathway, after a series of reactions, PUFAs produce lipid hydroperoxide (LOOH), 15-hydroperoxyeicosatetraenoic acid (15-HpETE), or 13-hydroperoxy-linoleic acid (13-HpODE), followed by the participation of transition metals, Hock rearrangement, and C-C → C-H migration. Then, alkanaldehyde, alkenaldehyde, and *γ*-alkenaldehyde are formed after fracture, and finally, 4-HNE is produced [34]. In the enzymatic pathway, according to the oxidized fatty acid (i.e., its length, number, and position of double bonds), the oxidation products are different in length, unsaturation, and substitution number, resulting in different secondary products. Fatty acids can be divided into two types according to the position of the first double bond from the methyl end: *ω*-3 and *ω*-6 fatty acids, which affect the possible position of cleavage and the corresponding cleavage products. The compound 4-HNE is mainly produced by the precursor substances, *ω*-6PUFAs (linolenic acid, *γ*-linolenic acid, and arachidonic acid) through 15-lipoxygenase (15-LOX) [35]. *ω*-3-PUFAs, including alpha-linolenic acid, eicosapentaenoic acid, and docosahexaenoic acid, can also be cleaved to produce many reactive aldehydes. Moreover, arachidonic acid can be oxidized by cyclooxygenase-2(COX-2) to reactive carbonyl compounds such as 4-oxo-2-nonenal, 4-hydroperoxy-2-nonenal, 4-hydroxy-2E, 6Z-dodecadienal, and trans-4-HNE [36]. Among these secondary products of aldehydes, 4-HNE is undoubtedly the most studied and is considered a useful biomarker for LPO [37].

### 3.2. Characteristics of 4-HNE

**Figure 2:**
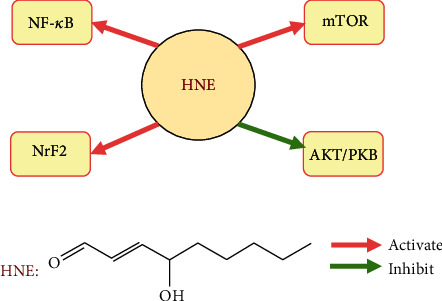
Summary of 4-hydroxy-2-nonenal (4-HNE) involvement in an aging-related signaling pathway. 4-HNE leading to NF-*κ*B, Nrf2, and mTOR pathway activation, and inhibiting AKT/PKB pathway activity.

4-HNE is considered one of the major mediators of oxidative stress in cells and tissues, that collectively lead to cell senescence by affecting the expression of various senescence-related signaling pathways [38] (such as NF-*κ*B, Nrf2, Akt/PKB, and mTOR), as shown in Figure 2. The combination of the two conjugated functional groups of 4-HNE, the carbonyl group (-C =0), and a double bond (C2/C3, -C=C-) facilitates the reaction of 4-HNE with biological molecules (including lipids, nucleic acids, and proteins) [39]. A mass spectrometry-based proteomic analysis of oxidative stress found that the electrophilic double bond and nucleophilic amino acid residues on proteins enable 4-HNE to form Michael adducts with lysine (Lys) [40], histidine (His) [41], and cysteine (Cys) [42] residues to increase the molecular masses of these amino acids by 156 Da (the molecular mass of HNE), or form Schiff-base adducts with its aldehyde group and Lys to an increase in the mass by 138 Da (Schiff-base formation with a net loss of water) [43, 44]. Moreover, the research of proteomic analysis of *in vitro* histone carbonylation sites showed that Schiff-base modification is labile and reversible, while that of Michael adducts are stable and non-reversible [45]. The Michael adducts can remain in cells for several hours before degradation, leading to the dysfunction of the targeted biomolecules. Therefore, the detection of HNE-biomolecular adducts is considered a valuable tool for evaluating various pathophysiological conditions associated with aging-related diseases.

## 4. Oxidative Stress, ALDH, and GSH

The human genome contains 19 known functional aldehyde dehydrogenase (ALDH) genes that are required for the biosynthesis of retinoic acid (RA) and other functional cellular or molecular regulators [46]. Previous mouse transplantation studies have demonstrated the role of ALDH as a molecular marker for hematopoietic stem cells (HSCs) and leukemic stem cells (LSCs). ALDH2 is the most active ALDH consisting of 517 amino acids to form 456 kDa subunits, each containing three domains. Recent studies have shown that ALDH2 plays a role as a stem cell marker and regulates cell functions related to self-renewal, expansion, differentiation, and resistance to drugs and radiation [47]. Moreover, ALDH is a key enzyme for the detoxification of endogenous and exogenous aldehyde substrates through NAD(P)^+^ dependent oxidation [48]. ALDH2 plays a role in the oxidative metabolism of toxic aldehydes in the brain, such as catecholaminergic metabolites (DOPAL and DOPEGAL) and 4-HNE [49].

Glutathione (GSH) is one of the most abundant low-molecular-weight mercaptans synthesized in cells. It is synthesized by adding Cys to glutamic acid and glycine. It plays a vital role in protecting cells from oxidative damage and toxicity of xenophilic reagents, as well as in the maintenance of redox homeostasis [50]. Lipids are one of the main substrates of glutathione-related protein (GSTM2), which can induce young cells to combine with 4-HNE, thereby reducing the content of 4-HNE in the liver, kidney, and serum of old mice [51]. Therefore, GSH plays a role in preventing aging-related diseases.

## 5. Physiological Role and Pathological Implications of Oxidative Stress derived 4-HNE

Oxidative stress leads to the production of LPO, which is one of the main sources of free radical-mediated damage that leads to the production of many secondary aldehydes, such as 4-HNE, 4-hydroxydodeca-(2E, 6Z)-dietary (4-HDDE), and 4-hydroxy-2E-hexenal (4-HHE) [52]. The mammalian brain is a highly oxidized organ [53], and PUFAs and relatively weak antioxidant defenses [54] render the brain vulnerable to free radical oxidative stress. Aging brain often has enhanced free radicals and decreased bioenergy [55]. Studies have shown that oxidative damage mediated by free radicals is closely related to the pathogenesis of aging-related neurodegenerative diseases [56–58]. The compound 4-HNE and the 4-HNE-protein complex are often detected in patients diagnosed with neurodegenerative diseases. Studies have shown that patients with AD, PD, HD, or ALS have increased levels of 4-HNE-protein adducts in their body fluid [59–61]. The 4-HNE-protein adducts can induce autoimmunity, and may be associated with the development of neurodegenerative diseases [62, 63]. Wang et al. [64] found a significant increase in anti-4-HNE-protein adduct antibody levels in MRL^+/+^ mice treated with trichloroethylene.

### 5.1. Oxidative Stress-derived 4-HNE and Alzheimer's disease (AD)

AD is a neurodegenerative disease with heterogeneous clinical symptoms, usually manifested as language, visuospatial, and executive dysfunctions [65]. Previous studies have suggested that AD is mainly caused by the amyloid plaques of amyloid-*β* (A*β*). However, all strategies and compounds for inhibiting A*β* deposition have failed in clinical trials [66], and the area with the highest deposition of A*β* does not coincide with the area with formation of neurofibrillary tangles (NFTs) and acute loss of synapses and neurons. Therefore, current focus of research has shifted to the hyperphosphorylated tau protein, which is affected by many pathological factors (such as abnormal activation of kinase, abnormal gene expression, and chronic stress), which is clinically known to cause excessive aggregation of NFTs [67]. There is also evidence that the brain tissue of AD patients is affected by toxic substances such as peroxides, alcohol, aldehydes, free carbonyls, and ketones produced by LPO due to oxidative stress. After the A*β* peptide is inserted into the neuronal lipid bilayer, it undergoes peroxidation to produce 4-HNE, which then covalently binds to the key neuronal membrane and cytosolic and mitochondrial proteins through a Michael addition reaction, leading to functional disorders of key neuronal proteins, neuronal death, and loss of cognitive ability [68].

The main neuronal protein binding sites for HNE are Lys, His, and Cys residues [69]. ApoE expression regulation and ApoE gene polymorphism play important roles in neurodegenerative diseases such as AD and PD, and other diseases [70]. The protein apoE2 has two Cys residues that can bind to 4-HNE, which can prevent neuronal protein damage. However, both Cys residues of ApoE4 could be replaced by arginine (Arg) residues, resulting in no 4-HNE binding to neuron proteins and leading to a cascade of events such as A*β* fibril deposition, A*β* oligomer production, neurofibril tangle formation, neuronal death, decreased synaptic plasticity associated with learning and memory, asymmetric lipid bilayer composition, loss of lipid homeostasis, and oxidative stress, which collectively increases the risk of AD [71, 72].

#### 5.1.1. ALDH and AD

A previous study [73] showed that accumulation of 4-HNE adducts, A*β* protein, and p-tau protein increased significantly in the hippocampus of ALDH2^−/−^ mice, indicating that ALDH2 significantly affected the AD process in mice. Joshi et al. [74] found that aldehyde dehydrogenase-2 deficiency (ALDH2∗2) reduced the clearance of toxic aldehydes, resulting in additional ROS production, followed by aldehyde addition to mitochondrial proteins, leading to mitochondrial dysfunction, ATP depletion, and ROS accumulation. These pathological changes in neurons and astrocytes lead to neuroinflammation, which affects the ability of glial cells to remove cell fragments. Therefore, activating ALDH2∗2 and increasing ALDH2-related compounds, such as Alda-1 (an activator of ALDH2 and ALDH2∗2), could be a potential therapeutic approach to slow down AD pathogenesis.

#### 5.1.2. GSH and AD

As an important cellular antioxidant, GSH balances the production and binding of free radicals and protects neurons from oxidative damage. It was observed that the level of GSH and the proportion of GSH/GSSG decreased in APP^NL-GF/NL-GF^ mice. After oral administration of GSH3, GSH levels increased in a dose-dependent manner and restored the ratio of GSH/GSSH. Concurrently, the level of 4-HNE in the mouse hippocampus decreased significantly [75], indicating that GSH can arrest the pathogenesis of AD by eliminating the toxic aldehyde, 4-HNE.

### 5.2. Oxidative Stress-derived 4-HNE and Parkinson's disease (PD)

PD is a major neurodegenerative disease. It is characterized by motor symptoms such as tremor, stiffness, muscular rigidity, and postural instability; and a series of non-motor symptoms, including autonomic and cognitive dysfunction [76]. The hallmark pathological features of PD are loss of dopaminergic neurons in the substantia nigra pars compacta (SNc) [77] and abnormal amyloid accumulation of misfolded *α*-synaptic protein (*α*-syn) in the formation of inclusions called Lewy bodies [78]. Studies have shown that the causes of dopaminergic neuronal death include impaired mitochondrial function [79] and dysbiosis of gut microbiota (imbalanced intestinal flora) [80].

Evidence has shown that high levels of oxidative stress in the SNc region of normal brain could be the triggering factor for a series of biochemical changes that lead to the death of dopaminergic cells. The early compensatory changes in dopamine caused by the degeneration of substantia nigra cells was found to increase oxidative stress and 4-HNE accumulation [81]. 4-HNE may affect PD in two main pathways. First, the complex formed following covalent modification of *α*-syn by 4-HNE (4-HNE/*α*-syn) tends to form more oligomers and fibrils than the simple *α*-syn, subsequently forming amyloid fibers that increase the toxicity to dopaminergic neurons [61, 82]. Zhang et al. [83] found that in neurons exposed to 4-HNE, the immunoreactivity of *α*-syn aggregates in whole neurites and cell bodies of different sizes, indicating that 4-HNE can induce the aggregation of *α*-syn in primary cortical neurons of rats.

The second pathway involves the destruction of the ubiquitin-proteasome system (UPS) by 4-HNE. UPS participates in the degradation of “bad” proteins related to damage, death, and modification by oxidation products such as 4-HNE [84]. However, the chemical structure, stability, and function of ubiquitin may be damaged by 4-HNE modification, thereby increasing cytotoxicity and affecting the process of PD. It was reported that 4-HNE can bind to the 26S proteasome [85], lead to the non-degradation of “bad” proteins and the accumulation of 4-HNE-protein complexes, hence affecting the pathology of PD. Additionally, some studies have shown that in addition to the direct relationship between 4-HNE/*α*-syn and dopaminergic transmission changes, 4-HNE can directly affect dopamine transmission by acting on dopamine receptors [86] and promotes the pathogenesis of PD.

#### 5.2.1. ALDH and PD

To verify the role of 4-HNE and other biogenic aldehydes in impaired detoxification homeostasis that leads to the pathogenesis of PD, Wey et al. [87] established an Aldh1a1−/− × Aldh2−/− mice PD model. The results of gait analysis and the accelerated rotation rod test indicated age-dependent motor performance defects: a significant decrease in the number of neurons immunoreactive to tyrosine hydroxylase (TH) in the substantia nigra, a decrease in dopamine and metabolites in the striatum, and an increase in neurotoxic bioaldehydes such as 4-HNE. These results support the hypothesis that damage to biological aldehyde detoxification mechanisms affects the pathophysiology of PD. Another study [88] determined the role of aldehyde dehydrogenase 1A1 (ALDH1A1) in mediating 4-HNE toxicity in PC12 cells using excessive ALDH1A1 and ALDH inhibitor disulfiram. Results showed that ALDH1A1 was downregulated in the brain tissue of patients with PD, 4-HNE toxicity was elevated, and ALDH activity was negatively correlated with the content of 4-HNE-protein adducts. Therefore, it is considered that the ALDH1A1 activator has a neuroprotective effect on patients with PD by reducing the content of 4-HNE-protein and inhibiting cytotoxicity [89, 90].

#### 5.2.2. Glutathione (GSH) and PD

Brain GSH levels in patients with PD are decreased, and the dopamine D2 receptor (DRD2) in astrocytes can regulate the synthesis of GSH through pyruvate kinase isozyme type M2 (PKM2)-mediated transactivation of Nrf2. In addition, pyridoxine can dimerize PKM2 to promote GSH biosynthesis [91]. Further experiments showed that pyridoxine supplementation increased the resistance of dopaminergic neurons in the substantia nigra to neurotoxicity in wild-type mice and astrocyte DRD2 conditional knockout mice. Thus, PKM2 may be a potential target for PD treatment.

### 5.3. Oxidative Stress derived 4-HNE and Cancer

ROS are involved in cancer-related cellular processes, such as proliferation, apoptosis, differentiation, cell migration, and DNA damage [92, 93]. However, due to their short half-lives, the carcinogenic effects of ROS are limited to the areas adjacent to their production. Nevertheless, the secondary product of LPO, 4-HNE is known to exert more complex carcinogenic effect on cell activity.

The carbonyl group on C1, the double bond carbon at the C3-C4 site, and the electron-absorbing hydroxyl group on C4 in 4-HNE further aggravate the electrophilicity of C3, which makes it easy for C3 to covalently bind to the primary amine of amino acid residues to form a Schiff-base modification [45]. The Schiff base produced by the covalent binding of 4-HNE-Lys forms a very stable pyrrole compound after cyclization. Pyrrole and its derivatives exhibit significant anticancer activities [94]. Therefore, 4-HNE can spread from the site of origin, change the structure and function of a corresponding protein, and lead to carcinogenesis. DNA damage plays an important role in mutagenesis, carcinogenesis, aging, and other pathophysiological conditions. 4-HNE can covalently bind to DNA, affecting the genomic function of normal cells, leading to carcinogenesis [95]. In addition to its direct cytotoxicity, 4-HNE is involved in regulating cellular signaling pathways, especially the Nrf2/Keap1/ARE pathways [96, 97]. Studies have shown that the distribution of 4-HNE in squamous cell carcinoma depends on the clinical stage and histological grade of these tumors [98]. Immunohistochemical analysis showed that the expression levels of 4-HNE in well, moderately, and poorly differentiated prostate cancer (PCa) were significantly higher than those in benign prostatic hyperplasia (BPH) tissue [99]. Therefore, the expression of 4-HNE is related to the grade of prostate cancer, making it a potential new biological reference marker for the prognosis of prostate cancer.

4-HNE plays a contrasting role in promoting cancer development and arresting tumor growth via upregulating the metabolic pathway of RLIP76 detoxification in tumors [96]. Low levels of 4-HNE can increase the differentiation markers of breast cancer stem cells (BCSCs). In contrast, high levels of chronic 4-HNE increase the concentration of GSH and Nrf2, hence increasing antioxidant protection [100].

#### 5.3.1. ALDH and Cancer

ALDH is considered a reliable marker of cancer stem cells (CSCs), which is widely used to enrich CSC subsets from various cell lines and solid tumors [101]. ALDH1 has three main isotypes, ALDH1A1, ALDH1A2, and ALDH1A3, which are involved in self-renewal, differentiation, and self-protection, and are markers of normal tissue stem cells (SCs) and cancer stem cells (CSCs) [48]. ALDH2 is a key enzyme that protects the heart from oxidative stress by consuming 4-HNE, and the metabolism of ROS and 4-HNE is thought to be deeply involved in cancer cell death. Hence, ALDH2 is considered to play an important role in cancer treatment [102].

#### 5.3.2. GSH and Cancer

The imbalanced redox homeostasis leads to an increased ROS content in tumor cells and cell death, which is an effective cancer treatment strategy. Studies have shown that GSH depletion and biosynthesis inhibition can reduce the concentration of GSH in cancer cells and enhance the therapeutic effect of photodynamic therapy (PDT) in cancer patients [103]. Moreover, the increased expression of GSH can protect cells from 4-HNE-induced cell damage and reduce cancer risk. It was reported that homocysteine (Hcy) induced the expression of Nrf2 protein and increased the expression of glutathione in HepG2 cells in a concentration-dependent manner, indicating that Hcy can induce GSH expression and mediate the antioxidant transcription factor Nrf2 to protect HepG2 cells from damage induced by the LPO secondary product, 4-HNE [104].

### 5.4. Oxidative Stress-derived 4-HNE and aging-related hearing loss (ARHL)

ARHL results from various factors, including aging, noise, ototoxic chemicals, heredity, epigenetic variables, and lifestyle. ROS accumulation and oxidative damage lead to abnormal cell function, damaged cell vitality, and eventually lead to functional decline and aging of the auditory system [105]. The compound 4-HNE is one of the most abundant end-products of LPO, which can lead to ARHL. A study showed that 4-HNE levels in aged deaf mice were higher than those in the young control group [106]. This is related to the decrease in spiral ganglion neuron (SGN) density and the thickness of hair cells and vascular stria in the cochlea of aged mice. Additionally, sublethal concentrations of H_2_O_2_ induced ROS, which then led to DNA damage. Following exposure to H_2_O_2_, mitotic cochlear implant cells showed the key characteristics of senescent cells, including significantly increased expression of p21, p38, and p-p38, decreased expression of p19 and BubR1, and positive labeling of *β*-galactosidase. It was suggested that the DNA damage response induced by ROS drives the senescence of cochlear cells and promotes the pathology of ARHL [107].

#### 5.4.1. GSH and ARHL

Glutathione transferase (GST), an important detoxifying enzyme, protects cells by catalyzing the binding of toxic compounds to reduced GSH. GSTA4 has a high catalytic effect on 4-HNE, and the combination of GSTA4 and GSH is considered the most effective means to eliminate 4-HNE. GSTA1 and GSTA2 can catalyze the reduction of fatty acid hydrogen peroxide (FA-OOH) and phospholipid hydrogen peroxide (PL-OOH), which are then reduced to the corresponding alcohol oxidized glutathione (GSSG) and water as by-products, thus preventing the formation of 4-HNE. GSTA4 and GSTA5 combine 4-HNE with GSH to form a GSH-4-HNE conjugate (GSH-4-HNE), which is then eliminated by the transmembrane transporters [9].

Cisplatin treatment was used to increase the level of 4-HNE in SGNs of WT female mice and the activity of GSTA4 on 4-HNE in the cochlea. In female GSTA4^−/−^ mice, cisplatin treatment increased the content of 4-HNE in cochlear neurons. In CBA/CaJ mice, ovariectomy decreased the mRNA expression of Gsta4 and the GSTA4 protein levels in the inner ear. Therefore, GSTA4-dependent detoxification may play a role in estrogen-mediated neuroprotection [108]. Moreover, GSTA4-mediated 4-HNE detoxification may play a role in protecting cochlear cell death, noise exposure, and age-related hearing loss [106].

### 5.5. Oxidative Stress-derived 4-HNE, aging-related macular degeneration (AMD), and dry eye

AMD is a disease that affects the macular region of the retina and can lead to a gradual loss of central vision. AMD is a multifactorial disease and its pathogenesis manifested as various disorders of the complement system and lipids, angiogenesis, inflammation, and extracellular matrix pathways [109]. Oxidative stress-induced retinal pigment epithelium (RPE) damage is considered a key factor in AMD pathology. Continuous exposure to oxidative stress in RPE cells can lead to the accumulation of damaged cellular proteins, lipids, nucleic acids, and organelles, including mitochondria [110], which aggravates AMD symptoms. RPE is rich in lipids, has high metabolic needs, is prone to LPO that accumulates 4-HNE, which made 4-HNE an ideal AMD retinal biomarker. Additionally, 4-HNE can induce apoptosis, lysosome imbalance, and lipofuscin production in RPE by activating a variety of molecules, such as NF-*κ*B, p53, Caspase-3, and NOX4, thus destroying the self-repair function of photoreceptor cells and causing AMD [111].

Oxidative stress and subsequent chronic inflammatory mediators can lead to the death of RPE cells, which is a therapeutic target for AMD. However, the molecular mechanism underlying the link between oxidative stress and inflammation remains unclear. A cytokine array was used to evaluate cytokine production in RPE induced by 4-HNE [112]. Molecular analysis confirmed that 4-HNE induced the production of IL-6, IL-1, and TNF-*α* by promoting the extracellular outflow of HSP70 and induced the production of low concentrations of IL-10 and TGF-*β*, thus playing a pro-inflammatory role in RPE cells.

Epidemiological studies have shown that the incidence of xerophthalmia increases with age, indicating an association with aging. In a dry eye model caused by continuous exposure to low humidity airflow for 30 days, the immunoreactive immune stress marker 4-HNE was found to increase with increased fluorescence, a clinical feature of epithelial lesions. In patients with xerophthalmia, it was found that the expression of the lipid peroxide marker 4-HNE on the surface membranes of eye increased compared to patients without xerophthalmia [113], suggesting that xerophthalmia is associated with an increased level of 4-HNE, a secondary product of oxidative stress.

#### 5.5.1. ALDH and AMD

A new model of RPE degeneration *in vivo* was established using spermidine as an inducer. Spermidine (20–30 nmol/eye) could destroy retinal electrophysiology and barrier function, causing degeneration of the retinal pigment epithelium and photoreceptors. On the 7th day after using ALDH (1.5 U/eye), the increase in permeability of blood-retinal barrier (BRB) induced by spermidine and the degeneration of RPE and photoreceptors were significantly inhibited. In addition, an acrolein-modified protein immunoassay was performed in RPE cells injected with spermidine. Results showed that ALDH could significantly inhibit oxidative stress-induced RPE degeneration [114].

#### 5.5.2. GSH and AMD

Cigarette smoking is the most important environmental risk factor for the occurrence of AMD, and damage to the RPE may be the cause of AMD. Exposure of RPE cells to cigarette smoke extract (CSE) or hydroquinone (HQ) leads to oxidative damage and apoptosis, characterized by cell size reduction and nuclear condensation. Evidence of oxidative damage also includes increased LPO (4-HNE) and mitochondrial superoxide production, and decreased intracellular GSH. Moreover, exogenous administration of the antioxidant GSH prevents oxidative damage of the RPE induced by cerebrospinal fluid [115].

### 5.6. Other Diseases linked to 4-HNE

The aldehyde compound 4-HNE forms adducts with free amino and mercaptan groups of proteins in the blood vessels causing the accumulation of 4-HNE adducts, which gradually leads to cell dysfunction, tissue damage, and atherosclerosis-related diseases. By forming the 4-HNE-apoB (apolipoprotein B) adducts, 4-HNE deviates from the low-density lipoprotein (LDL) metabolism involving scavenger receptor pathway of macrophages to the formation of foam cells, thus promoting atherogenesis. The 4-HNE adducts accumulate in the lipid necrotic core of advanced atherosclerotic lesions and may locally participate in macrophage and smooth muscle cell apoptosis, resulting in plaque instability and rupture, thus increasing the risk of atherosclerotic thrombosis events [116].

Immunohistochemistry and confocal immunofluorescence studies showed that 4-HNE-His adducts accumulated in the intima, media, and adventitia of the human aorta in an age-related manner and were mainly expressed in the smooth muscle cells. Therefore, the secondary product of LPO, especially 4-HNE, plays a complex role in elastin homeostasis, vascular wall remodeling, and atherosclerosis during aging [117].

Rossin et al. [118] found that 4-HNE and oxysterols exert their action intracellularly, by altering the redox balance of normal cells and activating antioxidant response signals. 4-HNE induces the cell signaling pathways of proliferation and survival which drive cells towards tumor resistance, developing colorectal cancer (CRC) and Inflammatory Bowel Disease (IBD).

## 6. Conclusions

**Figure 3:**
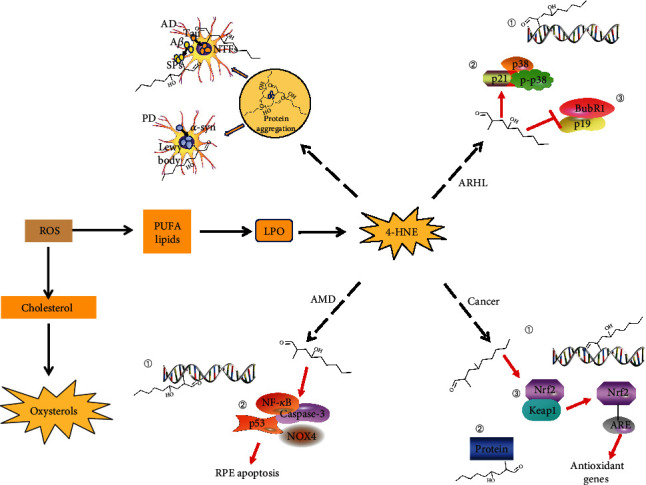
Role of 4-HNE in aging-related diseases. AD and PD: 4-HNE induces protein aggregation, abnormal aggregates of A*β* forms SP, and hyperphosphorylation of tau protein to form NFTs, which eventually leads to AD; aggregates of *α*-synuclein form Lewy body-like inclusions leading to PD. ARHL: 4-HNE (1) causes DNA damage; (2) increases p21, p38, and p-p38 activity; (3) reduces p19 and BubR1 activity. Cancer: 4-HNE (1) causes DNA damage; (2) affects protein function; (3) directly modifies cysteine residues (Cys 513, 518) on KEAP1, thus leading to KEAP1/Nrf2 pathway activation and increases the expression levels of Nrf2-ARE target genes, thus, activating the antioxidant-related pathways to influence cancer progression. AMD: 4-HNE (1) causes DNA damage; (2) activates NF-*κ*B, p53, Caspase-3, and NOX4 activity, thereby, inducing retinal pigment epithelium (RPE) apoptosis.

Toxic aldehydes such as 4-HNE, produced via LPO due to the accumulation of ROS, play an important role in the development of aging-related diseases (Figure 3 and Table 1). 4-HNE can covalently bind to the membranes of key neurons that leads to the formation and aggregation of neurofibrillary tangles, and ultimately results in neuronal protein dysfunction, death, cognitive impairment, language disorders, and other neurodegenerative symptoms that collectively aggravate the risk of AD and PD. The electrophilicity of C3 in 4-HNE causes 4-HNE to constitutively bind to proteins and DNA, and this tendency leads to the compromised genome and protein function that gives rise to tumorigenesis and pathogenesis of various aging-related diseases. Evidently, the accumulation of 4-HNE-protein adducts *in vivo* affects the regulation of Nrf2/Keap1/ARE signaling pathways. This review also found that the toxic effect of 4-HNE on lipoprotein is related to the formation of atherosclerosis, and its response to collagen may be the cause of cardiovascular tissue sclerosis. 4-HNE can activate various molecules, such as NF-*κ*B and NOX4, to induce RPE apoptosis, lysosomal imbalance, and lipofuscin production, resulting in photoreceptor cell destruction and consequently, age-related visual impairment such as AMD. Studies have found that ALDH and GSH pathways have a potential detoxification effect on toxic aldehydes by delaying the development of aging-related diseases caused by 4-HNE. Therefore, 4-HNE may be a potential target for clinical therapy aiming at delaying aging-related diseases.

## Figures and Tables

**Table 1:** Pharmacological measures of 4-HNE detoxification.

Disease	Protein	Detoxification	Reference
AD	apoE (Cys)	apoE (Cys)-4-HNE prevents neuronal protein damage	[70–72]
	Alda-1	Activate ALDH2 and ALDH2∗2	[74]
	GSH	Eliminate the toxic aldehyde	[75]

PD	ALDHA1	Reduce the content of 4-HNE-protein and inhibiting cytotoxicity	[89, 90]
	PKM2	Regulate the synthesis of GSH	[91]

Cancer	ALDH2	High levels of 4-HNE increase GSH and NRF2	[100]
		Consume 4-HNE	[102]
	Hcy	Induce GSH mediate Nrf2 protect HepG2	[104]

ARHL	GSTA1, GSTA2	Prevent 4-HNE formation	[9]
	GSTA4, GSTA5	GSH-4-HNE	[9]

AMD	ALDH	Inhibit retinal epithelial degeneration	[114]
	GSH	Prevent oxidative damage of RPE	[115]

## Data Availability

The data used to support the findings of this study are included within the article.

## References

[1] Davalli P., Mitic T., Caporali A., Lauriola A., D’Arca D. (2016). ROS, cell senescence, and novel molecular mechanisms in aging and age-related diseases. *Oxidative Medicine and Cellular Longevity*.

[2] Islam M. T. (2017). Oxidative stress and mitochondrial dysfunction-linked neurodegenerative disorders. *Neurological Research*.

[3] Hauck A. K., Huang Y., Hertzel A. V., Bernlohr D. A. (2019). Adipose oxidative stress and protein carbonylation. *The Journal of Biological Chemistry*.

[4] Yu Y., Cui Y., Niedernhofer L. J., Wang Y. (2016). Occurrence, biological consequences, and human health relevance of oxidative stress-induced DNA damage. *Chemical Research in Toxicology*.

[5] Sottero B., Rossin D., Poli G., Biasi F. (2018). Lipid oxidation products in the pathogenesis of inflammation-related gut diseases. *Current Medicinal Chemistry*.

[6] Zarrouk A., Vejux A., Mackrill J. (2014). Involvement of oxysterols in age-related diseases and ageing processes. *Ageing Research Reviews*.

[7] Hou Y., Dan X., Babbar M. (2019). Ageing as a risk factor for neurodegenerative disease. *Nature Reviews. Neurology*.

[8] Raffaghello L., Longo V. (2017). Metabolic alterations at the crossroad of aging and oncogenesis. *International Review of Cell and Molecular Biology*.

[9] Someya S., Kim M. J. (2021). Cochlear detoxification: role of alpha class glutathione transferases in protection against oxidative lipid damage, ototoxicity, and cochlear aging. *Hearing Research*.

[10] Brouilette S. W., Moore J. S., McMahon A. (2007). Telomere length, risk of coronary heart disease, and statin treatment in the west of Scotland primary prevention study: a nested case-control study. *Lancet*.

[11] Chen S. J., Lin T. B., Peng H. Y. (2021). Cytoprotective Potential of Fucoxanthin in Oxidative Stress-Induced Age-Related Macular Degeneration and Retinal Pigment Epithelial Cell Senescence In Vivo and In Vitro. *Marine Drugs*.

[12] de Souza R. G., Yu Z., Hernandez H. (2021). Modulation of oxidative stress and inflammation in the aged lacrimal gland. *The American Journal of Pathology*.

[13] Xiao H., Jedrychowski M. P., Schweppe D. K. (2020). A Quantitative Tissue-Specific Landscape of Protein Redox Regulation during Aging. *Cell*.

[14] Zhang T., Gaffrey M. J., Li X., Qian W. J. (2021). Characterization of cellular oxidative stress response by stoichiometric redox proteomics. *American Journal of Physiology. Cell Physiology*.

[15] El Sayed N. S., Ghoneum M. H. (2020). Antia, a natural antioxidant product, attenuates cognitive dysfunction in Streptozotocin-induced mouse model of sporadic Alzheimer's disease by targeting the Amyloidogenic, inflammatory, autophagy, and oxidative stress pathways. *Oxidative Medicine and Cellular Longevity*.

[16] He L., He T., Farrar S., Ji L., Liu T., Ma X. (2017). Antioxidants maintain cellular redox homeostasis by elimination of reactive oxygen species. *Cellular Physiology and Biochemistry*.

[17] Gulçin İ., Taslimi P., Aygün A. (2018). Antidiabetic and antiparasitic potentials: Inhibition effects of some natural antioxidant compounds on *α*-glycosidase, *α*-amylase and human glutathione *S* -transferase enzymes. *International Journal of Biological Macromolecules*.

[18] Gaucher C., Boudier A., Bonetti J., Clarot I., Leroy P., Parent M. (2018). Glutathione: Antioxidant Properties Dedicated to Nanotechnologies. *Antioxidants (Basel)*.

[19] Moldogazieva N. T., Mokhosoev I. M., Mel’nikova T. I., Porozov Y. B., Terentiev A. A. (2019). Oxidative stress and advanced Lipoxidation and glycation end products (ALEs and AGEs) in aging and age-related diseases. *Oxidative Medicine and Cellular Longevity*.

[20] Desai K. M., Chang T., Wang H. (2010). Oxidative stress and aging: is methylglyoxal the hidden enemy?. *Canadian Journal of Physiology and Pharmacology*.

[21] Su L. J., Zhang J. H., Gomez H. (2019). Reactive oxygen species-induced lipid peroxidation in apoptosis, autophagy, and Ferroptosis. *Oxidative Medicine and Cellular Longevity*.

[22] Bayır H., Anthonymuthu T. S., Tyurina Y. Y. (2020). Achieving life through death: redox biology of lipid peroxidation in Ferroptosis. *Cell Chemical Biology*.

[23] Ursini F., Maiorino M. (2020). Lipid peroxidation and ferroptosis: the role of GSH and GPx4. *Free Radical Biology & Medicine*.

[24] Ayala A., Muñoz M. F., Argüelles S. (2014). Lipid peroxidation: production, metabolism, and signaling mechanisms of malondialdehyde and 4-hydroxy-2-nonenal. *Oxidative Medicine and Cellular Longevity*.

[25] Del Rio D., Stewart A. J., Pellegrini N. (2005). A review of recent studies on malondialdehyde as toxic molecule and biological marker of oxidative stress. *Nutrition, Metabolism, and Cardiovascular Diseases*.

[26] Tsikas D. (2017). Assessment of lipid peroxidation by measuring malondialdehyde (MDA) and relatives in biological samples: analytical and biological challenges. *Analytical Biochemistry*.

[27] Balogh E., Veale D. J., McGarry T. (2018). Oxidative stress impairs energy metabolism in primary cells and synovial tissue of patients with rheumatoid arthritis. *Arthritis Research & Therapy*.

[28] Aksu N., Samadi A., Yalçınkaya A. (2020). Evaluation of oxysterol levels of patients with silicosis by LC-MS/MS method. *Molecular and Cellular Biochemistry*.

[29] Rašić I., Rašić A., Akšamija G., Radović S. (2018). The relationship between serum level of malondialdehyde and progression of colorectal cancer. *Acta Clinica Croatica*.

[30] Shrivastava A., Mishra S. P., Pradhan S. (2021). An assessment of serum oxidative stress and antioxidant parameters in patients undergoing treatment for cervical cancer. *Free Radical Biology & Medicine*.

[31] Pandey S., Singh B., Yadav S. K., Mahdi A. A. (2018). Novel biomarker for neurodegenerative diseases- motor neuron disease (MND), cerebellar ataxia (CA) and Parkinson's disease (PD). *Clinica Chimica Acta*.

[32] Kim M., Kim M., Yoo H. J., Sun Y., Lee S. H., Lee J. H. (2018). PPARDrs7770619 polymorphism in a Korean population: association with plasma malondialdehyde and impaired fasting glucose or newly diagnosed type 2 diabetes. *Diabetes & Vascular Disease Research*.

[33] Pecorelli A., Woodby B., Prieux R., Valacchi G. (2019). Involvement of 4-hydroxy-2-nonenal in pollution-induced skin damage. *BioFactors*.

[34] Sousa B. C., Pitt A. R., Spickett C. M. (2017). Chemistry and analysis of HNE and other prominent carbonyl-containing lipid oxidation compounds. *Free Radical Biology & Medicine*.

[35] Breitzig M., Bhimineni C., Lockey R., Kolliputi N. (2016). 4-Hydroxy-2-nonenal: a critical target in oxidative stress?. *American Journal of Physiology. Cell Physiology*.

[36] Wang X., Allen T. D., Yang Y., Moore D. R., Huycke M. M. (2013). Cyclooxygenase-2 generates the endogenous mutagen trans-4-hydroxy-2-nonenal in enterococcus faecalis-infected macrophages. *Cancer Prevention Research (Philadelphia, Pa.)*.

[37] Chapple S. J., Cheng X., Mann G. E. (2013). Effects of 4-hydroxynonenal on vascular endothelial and smooth muscle cell redox signaling and function in health and disease. *Redox Biology*.

[38] Zhang H., Forman H. J. (2017). 4-hydroxynonenal-mediated signaling and aging. *Free Radical Biology & Medicine*.

[39] Zhang H., Lyn N., Haghani A., Forman H. J. (2020). Detection of HNE modification of proteins in aging mouse tissues: a Western blot-based approach. *Methods in Molecular Biology*.

[40] Dantas L. S., Viviani L. G., Inague A. (2020). Lipid aldehyde hydrophobicity affects apo-SOD1 modification and aggregation. *Free Radical Biology & Medicine*.

[41] Lee S. H., Matsunaga A., Oe T. (2018). Inhibition effect of pyridoxamine on lipid hydroperoxide-derived modifications to human serum albumin. *PLoS One*.

[42] Alviz-Amador A., Galindo-Murillo R., Pineda-Alemán R. (2018). Development and benchmark to obtain AMBER parameters dataset for non-standard amino acids modified with 4-hydroxy-2-nonenal. *Data in Brief*.

[43] Carini M., Aldini G., Facino R. M. (2004). Mass spectrometry for detection of 4-hydroxy-trans-2-nonenal (HNE) adducts with peptides and proteins. *Mass Spectrometry Reviews*.

[44] Aslebagh R., Pfeffer B. A., Fliesler S. J., Darie C. C. (2016). Mass spectrometry-based proteomics of oxidative stress: identification of 4-hydroxy-2-nonenal (HNE) adducts of amino acids using lysozyme and bovine serum albumin as model proteins. *Electrophoresis*.

[45] Hauck A. K., Zhou T., Upadhyay A. (2020). Histone Carbonylation Is a Redox-Regulated Epigenomic Mark That Accumulates with Obesity and Aging. *Antioxidants (Basel)*.

[46] Yang X., Yao R., Wang H. (2018). Update of ALDH as a potential biomarker and therapeutic target for AML. *BioMed Research International*.

[47] Vassalli G. (2019). Aldehyde dehydrogenases: not just markers, but functional regulators of stem cells. *Stem Cells International*.

[48] Tomita H., Tanaka K., Tanaka T., Hara A. (2016). Aldehyde dehydrogenase 1A1 in stem cells and cancer. *Oncotarget*.

[49] Deza-Ponzio R., Herrera M. L., Bellini M. J., Virgolini M. B., Hereñú C. B. (2018). Aldehyde dehydrogenase 2 in the spotlight: the link between mitochondria and neurodegeneration. *Neurotoxicology*.

[50] Forman H. J., Zhang H., Rinna A. (2009). Glutathione: overview of its protective roles, measurement, and biosynthesis. *Molecular Aspects of Medicine*.

[51] Fafián-Labora J. A., Rodríguez-Navarro J. A., O'Loghlen A. (2020). Small Extracellular Vesicles Have GST Activity and Ameliorate Senescence-Related Tissue Damage. *Cell Metabolism*.

[52] Bacot S., Bernoud-Hubac N., Baddas N. (2003). Covalent binding of hydroxy-alkenals 4-HDDE, 4-HHE, and 4-HNE to ethanolamine phospholipid subclasses. *Journal of Lipid Research*.

[53] Erecińska M., Silver I. A. (2001). Tissue oxygen tension and brain sensitivity to hypoxia. *Respiration Physiology*.

[54] Cobley J. N., Fiorello M. L., Bailey D. M. (2018). 13 reasons why the brain is susceptible to oxidative stress. *Redox Biology*.

[55] Bayliak M. M., Sorochynska O. M., Kuzniak O. V. (2021). Middle age as a turning point in mouse cerebral cortex energy and redox metabolism: modulation by every-other-day fasting. *Experimental Gerontology*.

[56] Bascuas T., Kropp M., Harmening N., Asrih M., Izsvák Z., Thumann G. (2020). Induction and analysis of oxidative stress in <em>Sleeping Beauty</em> transposon-transfected human retinal pigment epithelial cells. *Journal of Visualized Experiments*.

[57] Garrido-Pascual P., Alonso-Varona A., Castro B., Burón M., Palomares T. (2020). Hydrogen Peroxide-Preconditioned Human Adipose-Derived Stem Cells Enhance the Recovery of Oligodendrocyte-Like Cells after Oxidative Stress-Induced Damage. *International Journal of Molecular Sciences*.

[58] Giordano S., Darley-Usmar V., Zhang J. (2014). Autophagy as an essential cellular antioxidant pathway in neurodegenerative disease. *Redox Biology*.

[59] Shibata N., Kato Y., Inose Y. (2011). 4-Hydroxy-2-nonenal upregulates and phosphorylates cytosolic phospholipase a(2) in cultured Ra2 microglial cells via MAPK pathways. *Neuropathology*.

[60] Di Domenico F., Tramutola A., Butterfield D. A. (2017). Role of 4-hydroxy-2-nonenal (HNE) in the pathogenesis of alzheimer disease and other selected age-related neurodegenerative disorders. *Free Radical Biology & Medicine*.

[61] Sinha M. S., Giraldo A. M. V., Öllinger K., Hallbeck M., Civitelli L. (2018). Lipid vesicles affect the aggregation of 4-hydroxy-2-nonenal-modified *α*-synuclein oligomers. *Biochimica et Biophysica Acta (BBA)-Molecular Basis of Disease*.

[62] De Virgilio A. (2016). Parkinson's disease: autoimmunity and neuroinflammation. *Autoimmunity Reviews*.

[63] Sabatino J. J., Pröbstel A. K., Zamvil S. S. (2019). B cells in autoimmune and neurodegenerative central nervous system diseases. *Nature Reviews. Neuroscience*.

[64] Wang G., Wakamiya M., Wang J., Ansari G. A. S., Firoze Khan M. (2015). iNOS null MRL+/+ mice show attenuation of trichloroethene-mediated autoimmunity: contribution of reactive nitrogen species and lipid-derived reactive aldehydes. *Free Radical Biology & Medicine*.

[65] Atri A. (2019). The Alzheimer's disease clinical Spectrum: diagnosis and management. *The Medical Clinics of North America*.

[66] Zhang H., Zheng Y. (2019). Amyloid Hypothesis in Alzheimer's Disease:Pathogenesis,Prevention, and Management. *Zhongguo Yi Xue Ke Xue Yuan Xue Bao*.

[67] Gao Y., Tan L., Yu J. T., Tan L. (2018). Tau in Alzheimer's disease: mechanisms and therapeutic strategies. *Current Alzheimer Research*.

[68] Butterfield D. A. (2020). Brain lipid peroxidation and alzheimer disease: synergy between the Butterfield and Mattson laboratories. *Ageing Research Reviews*.

[69] Alviz-Amador A., Galindo-Murillo R., Pineda-Alemán R. (2019). 4-HNE carbonylation induces local conformational changes on bovine serum albumin and thioredoxin. A molecular dynamics study. *Journal of Molecular Graphics & Modelling*.

[70] Yin Y., Wang Z. (2018). ApoE and neurodegenerative diseases in aging. *Advances in Experimental Medicine and Biology*.

[71] Butterfield D. A., Mattson M. P. (2020). Apolipoprotein E and oxidative stress in brain with relevance to Alzheimer's disease. *Neurobiology of Disease*.

[72] Butterfield D. A., Boyd-Kimball D. (2018). Oxidative stress, amyloid-*β* peptide, and altered key molecular pathways in the pathogenesis and progression of Alzheimer's disease. *Journal of Alzheimer's Disease*.

[73] D'Souza Y. (2015). Characterization of Aldh2 (-/-) mice as an age-related model of cognitive impairment and Alzheimer's disease. *Molecular Brain*.

[74] Joshi A. U., van Wassenhove L. D., Logas K. R. (2019). Aldehyde dehydrogenase 2 activity and aldehydic load contribute to neuroinflammation and Alzheimer's disease related pathology. *Acta Neuropathologica Communications*.

[75] Izumi H., Sato K., Kojima K., Saito T., Saido T. C., Fukunaga K. (2020). Oral glutathione administration inhibits the oxidative stress and the inflammatory responses in App^NL−G-F/NL−G-F^ knock-in mice. *Neuropharmacology*.

[76] Balestrino R., Schapira A. H. V. (2020). Parkinson disease. *European Journal of Neurology*.

[77] Surmeier D. J. (2018). Determinants of dopaminergic neuron loss in Parkinson's disease. *The FEBS Journal*.

[78] Rocha E. M., De Miranda B., Sanders L. H. (2018). Alpha-synuclein: Pathology, mitochondrial dysfunction and neuroinflammation in Parkinson's disease. *Neurobiology of Disease*.

[79] Bose A., Beal M. F. (2016). Mitochondrial dysfunction in Parkinson's disease. *Journal of Neurochemistry*.

[80] Sun M. F., Shen Y. Q. (2018). Dysbiosis of gut microbiota and microbial metabolites in Parkinson's disease. *Ageing Research Reviews*.

[81] Jenner P. (2003). Oxidative stress in Parkinson's disease. *Annals of Neurology: Official Journal of the American Neurological Association and the Child Neurology Society*.

[82] Bae E. J., Ho D. H., Park E. (2013). Lipid peroxidation product 4-hydroxy-2-nonenal promotes seeding-capable oligomer formation and cell-to-cell transfer of *α*-synuclein. *Antioxidants & Redox Signaling*.

[83] Zhang S., Eitan E., Wu T. Y., Mattson M. P. (2018). Intercellular transfer of pathogenic *α*-synuclein by extracellular vesicles is induced by the lipid peroxidation product 4-hydroxynonenal. *Neurobiology of Aging*.

[84] Wang Y., Le W. D. (2019). Autophagy and ubiquitin-proteasome system. *Advances in Experimental Medicine and Biology*.

[85] Grasso G., Axelsen P. H. (2017). Effects of covalent modification by 4-hydroxy-2-nonenal on the noncovalent oligomerization of ubiquitin. *Journal of Mass Spectrometry*.

[86] Shin Y., White B. H., Uh M., Sidhu A. (2003). Modulation of D1-like dopamine receptor function by aldehydic products of lipid peroxidation. *Brain Research*.

[87] Wey M. C., Fernandez E., Martinez P. A., Sullivan P., Goldstein D. S., Strong R. (2012). Neurodegeneration and motor dysfunction in mice lacking cytosolic and mitochondrial aldehyde dehydrogenases: implications for Parkinson's disease. *PLoS One*.

[88] Kong D., Kotraiah V. (2012). Modulation of aldehyde dehydrogenase activity affects (±)-4-hydroxy-2E-nonenal (HNE) toxicity and HNE-protein adduct levels in PC12 cells. *Journal of Molecular Neuroscience*.

[89] Tapias V., McCoy J. L., Greenamyre J. T. (2019). Phenothiazine normalizes the NADH/NAD^+^ ratio, maintains mitochondrial integrity and protects the nigrostriatal dopamine system in a chronic rotenone model of Parkinson's disease. *Redox Biology*.

[90] Yang L., Lin X., Tang H. (2020). Mitochondrial DNA mutation exacerbates female reproductive aging via impairment of the NADH/NAD^+^ redox. *Aging Cell*.

[91] Wei Y., Lu M., Mei M. (2020). Pyridoxine induces glutathione synthesis via PKM2-mediated Nrf2 transactivation and confers neuroprotection. *Nature Communications*.

[92] Moloney J. N., Cotter T. G. (2018). ROS signalling in the biology of cancer. *Seminars in Cell & Developmental Biology*.

[93] Klaunig J. E. (2018). Oxidative stress and cancer. *Current Pharmaceutical Design*.

[94] Adel M., Serya R. A. T., Lasheen D. S., Abouzid K. A. M. (2018). Pyrrolopyrimidine, a multifaceted scaffold in cancer targeted therapy. *Drug Research*.

[95] Faisal M., Shahab U., Alatar A. A., Ahmad S. (2017). Preferential recognition of auto-antibodies against 4-hydroxynonenal modified DNA in the cancer patients. *Journal of Clinical Laboratory Analysis*.

[96] Gasparovic A. C., Milkovic L., Sunjic S. B., Zarkovic N. (2017). Cancer growth regulation by 4-hydroxynonenal. *Free Radical Biology & Medicine*.

[97] Guéraud F. (2017). 4-Hydroxynonenal metabolites and adducts in pre-carcinogenic conditions and cancer. *Free Radical Biology & Medicine*.

[98] Jakovčević A., Žarković K., Jakovčević D. (2020). The Appearance of 4-Hydroxy-2-Nonenal (HNE) in Squamous Cell Carcinoma of the Oropharynx. *Molecules*.

[99] Song X., Wang R., Zhang S. X., Gong M., Zhang Y., Huang J. Y. (2020). Overexpressed HSF1 and 4-HNE relate to the malignant phenotype of prostate cancer. *Zhonghua Nan Ke Xue*.

[100] Čipak Gašparović A., Milković L., Dandachi N. (2019). Chronic Oxidative Stress Promotes Molecular Changes Associated with Epithelial Mesenchymal Transition, NRF2, and Breast Cancer Stem Cell Phenotype. *Antioxidants*.

[101] Toledo-Guzmán M. E., Hernández M. I., Gómez-Gallegos Á. A., Ortiz-Sánchez E. (2019). ALDH as a stem cell marker in solid tumors. *Current Stem Cell Research & Therapy*.

[102] Wang L. S., Wu Z. X. (2019). ALDH2 and cancer therapy. *Advances in Experimental Medicine and Biology*.

[103] Hu J., Wang T., Zhou L., Wei S. (2020). A ROS responsive nanomedicine with enhanced photodynamic therapy via dual mechanisms: GSH depletion and biosynthesis inhibition. *Journal of Photochemistry and Photobiology. B*.

[104] Zhang B., Dong J. L., Chen Y. L. (2017). Nrf2 mediates the protective effects of homocysteine by increasing the levels of GSH content in HepG2 cells. *Molecular Medicine Reports*.

[105] Bermúdez-Muñoz J. M., Celaya A. M., Hijazo-Pechero S., Wang J., Serrano M., Varela-Nieto I. (2020). G6PD overexpression protects from oxidative stress and age-related hearing loss. *Aging Cell*.

[106] Park H. J., Kim M. J., Han C. (2020). Effects of *Gsta4* deficiency on age-related cochlear pathology and hearing loss in mice. *Experimental Gerontology*.

[107] Benkafadar N., François F., Affortit C. (2019). ROS-induced activation of DNA damage responses drives senescence-like state in Postmitotic Cochlear cells: implication for hearing preservation. *Molecular Neurobiology*.

[108] Park H. J., Kim M. J., Rothenberger C. (2019). GSTA4 mediates reduction of cisplatin ototoxicity in female mice. *Nature Communications*.

[109] Mitchell P., Liew G., Gopinath B., Wong T. Y. (2018). Age-related macular degeneration. *Lancet*.

[110] Kaarniranta K., Uusitalo H., Blasiak J. (2020). Mechanisms of mitochondrial dysfunction and their impact on age-related macular degeneration. *Progress in Retinal and Eye Research*.

[111] Bae D., Gautam J., Jang H. (2018). Protective effects of 6-ureido/thioureido-2,4,5-trimethylpyridin-3-ols against 4-hydroxynonenal-induced cell death in adult retinal pigment epithelial-19 cells. *Bioorganic & Medicinal Chemistry Letters*.

[112] Yang H. J., Hu R., Sun H., Li X., Chen J. B. (2019). 4-HNE induces proinflammatory cytokines of human retinal pigment epithelial cells by promoting extracellular efflux of HSP70. *Experimental Eye Research*.

[113] Seen S., Tong L. (2018). Dry eye disease and oxidative stress. *Acta Ophthalmologica*.

[114] Ohashi K., Kageyama M., Shinomiya K. (2017). Spermidine oxidation-mediated degeneration of retinal pigment epithelium in rats. *Oxidative Medicine and Cellular Longevity*.

[115] Bertram K. M., Baglole C. J., Phipps R. P., Libby R. T. (2009). Molecular regulation of cigarette smoke induced-oxidative stress in human retinal pigment epithelial cells: implications for age-related macular degeneration. *American Journal of Physiology. Cell Physiology*.

[116] Nègre-Salvayre A., Garoby-Salom S., Swiader A., Rouahi M., Pucelle M., Salvayre R. (2017). Proatherogenic effects of 4-hydroxynonenal. *Free Radical Biology & Medicine*.

[117] Zarkovic K., Larroque-Cardoso P., Pucelle M. (2015). Elastin aging and lipid oxidation products in human aorta. *Redox Biology*.

[118] Rossin D., Calfapietra S., Sottero B., Poli G., Biasi F. (2017). HNE and cholesterol oxidation products in colorectal inflammation and carcinogenesis. *Free Radical Biology & Medicine*.

